# Health Care Needs and Services for Elder and Disabled Population: Findings from a Barcelona Study

**DOI:** 10.3390/ijerph17218071

**Published:** 2020-11-02

**Authors:** Jessica Rodriguez-Pereira, Jesica de Armas, Lorenzo Garbujo, Helena Ramalhinho

**Affiliations:** 1 Department Economics and Business, Universitat Pompeu Fabra, 08005 Barcelona, Spain; jesica.dearmas@upf.edu (J.d.A.); lorenzo.garbujo@alum.upf.edu (L.G.); helena.ramalhinho@upf.edu (H.R.); 2 Barcelona School of Management, Universitat Pompeu Fabra, 08008 Barcelona, Spain

**Keywords:** elder population, disabled population, health care services, descriptive analytics

## Abstract

Health care is a pillar of modern society. This study focuses on the use of descriptive analytics to provide demographic and territorial insights that will be of strategic importance in planning subsequent projects meant to improve health care services. We especially focus on the assessment of the elder and disabled population health care needs in Barcelona, and evaluate to what extent the current health care infrastructure is successful in covering the demand of these fragile population segment. This work is developed around three main assessments in the municipality of Barcelona: the elder and disabled health care demand, the available health care services, and the relationship between demand and services, showing that territorial and demographic aspects are relevant in assessing the health needs of the population.

## 1. Introduction

The relevance of planning in healthcare is indisputable. Complexity in health care processes is high, services are intertwined and involve many disciplines, making structural flexibility an important requirement. Each patient possesses their own individual characteristics that must be considered to offer the best care service, complicating process standardization. The result is that the healthcare domain represents unique challenges from a business process perspective [1].

Health care is a pillar of modern society and a fundamental social right. Access to healthcare should be effective for each person and costs should not prevent patients to receive the treatment they need. Moreover, preventive cure should also be available without inequalities. In truth, this scenario is still far from being the norm, as can be noted from key European Commission documents [2]. The reduction of inequalities is being pursued across Europe and new ways of delivering health care services have occured throughout the years. Nowadays, a paradigm shift in the way health care is delivered is taking place; society is shifting from a hospital-based model to a community and home-based model for health care with the home as a central place to receive treatment [3].

This work aims to assess Barcelona’s current health care infrastructure in relation to the elderly and disabled population. Barcelona aspires to become a benchmark for person-centered care through the deployment of specific home health care projects. Catalonia and Barcelona, in particular, have a large health services network and infrastructure that has been the object of several studies [4,5,6,7,8]. The Barcelona 2016–2020 Health Plan, created by the city’s Sanitary Consortium [9], includes the implementation of a comprehensive care model focused on people with chronic illnesses and complex needs, modernization of the old welfare paradigm and functional reformulation of healthcare teams to address complex needs, development of common guidelines and transversal practices meant to facilitate an integrated care model, enhancing palliative services and mental care. Furthermore, two other major objectives are formulated: the reduction of inequalities in health prioritizing actions in the neighborhoods characterized by the worst socioeconomic and health indicators and the exploitation digital tools to improve the coordination, transparency, monitoring and sharing of resources.

Innovation in health care practices is also the objective of two other major programs of Barcelona’s municipality. PERIS 2016–2020 is the strategic plan for health research and innovation and it is aligned with overall 2016–2020 Health Plan, stressing the importance of research as a fundamental value for improving health. The PERIS plan states that society is moving towards a health system based on the principles of scientific evidence-based medicine, personalization of interventions and bioethics and that research is key to face these challenges [10]. To support innovation, Catalonia launched the Public Data Analysis for Health Research and Innovation Programme in Catalonia (PADRIS program), which is meant to facilitate the reuse of health data to drive research. The European Health Commission sees the reuse of health information as a critical element for the planning and improving of health systems and PADRIS is meant to provide access to anonymized health information [11]. Thus, our objective is to identify and locate the needs of the elderly and disabled population, the current available infrastructure and the relationship between the two, in order to find patterns or weaknesses that are useful for the decision makers in the administration of health care services.

## 2. Materials and Methods

Analytics are driving innovation in the field and are shaping new perspectives on how to improve home health care [12]. For this reason, this study focuses on the use of descriptive analytics to provide demographic and territorial insights that will be of strategic importance in planning subsequent projects meant to improve care services. The analysis is developed for Barcelona municipality, although the methodology can be applied to any other area. The research conducted in this work has been developed around three main assessments in the municipality of Barcelona: the elderly and disabled population as a segment population with high health care demand, the available health care services, and the relationship between demand and services. Thereby, we provide an assessment on the population health care needs focused on the elderly and disabled population and evaluate to which extent the current health care infrastructure is successful in covering their demand. This specific population have greater special healthcare needs than the general population, such as for example, the ones discussed about preventive health services [13] or home health care for elder population [14,15,16,17,18,19], or access to general health services [20,21,22].

The demand assessment aims to profile the neighborhoods through their elder and disabled population. The idea is to segment the neighborhoods based on their inhabitants’ health characteristics to understand how the studied segment of population is distributed across the city. The goal is to detect major patterns that could later be exploited for process optimization. This is achieved through data analysis, involving descriptive analytics and relying on multivariate analysis techniques [23].

The later assessment of the organizational areas designed by the Sanitary Consortium of Barcelona has allowed us to compare and evaluate if such services are distributed matching the territorial needs and following certain parameters. These organizational areas to manage the various entities and actors that provide care services in Barcelona are the “Àrees Integrals de Salut” (AIS, Integral Health Areas) and the “Programa d’Atenció Domiciliària” (PADES, Home Care Program). Thus, the allocation of health care resources in relation to population distribution is examined and analyzed at different granularity levels: first by considering the singular neighborhood, then at PADES area level, and lastly from a high scope where population and resources are aggregated by AIS. The same kind of analytics are applied in this case.

The starting point to develop these three assessments is obviously collecting the data about demographics and health care services. Then, we performed a data exploration, and we have created frequency and contingency tables of the various datasets. After having filtered and standardized the data to match the aggregation level of interest, we have applied the various analysis techniques listed in the next sections to get the results. To this end, we have used different software and programming languages, e.g., SPSS, Phyton and R. Additionally, both the demographics characteristics and services locations are visually represented through the publicly available map generator software Instamap [24].

It is noteworthy that this methodology has been followed with the aim of painting a global picture of the health care in the city, and is meant to be an auxiliary tool of strategic importance for further developments to improve care services.

The used datasets and data analysis techniques are detailed below.

### 2.1. Datasets

We have collected the data from various open sources. Most of the datasets were obtained from the websites of Barcelona City Council and Area Metropolitana de Barcelona [25]. In particular, the Open Data BCN [26], a public resource promoted by the City Council offering plenty of open data for the common good, provides information on: neighborhoods’ geographical coordinates, hospitals and primary health services, elderly day care hospitalized centres, elderly hospitalized care homes, social centres with health services, population by age and by neighborhood, and disabled population by level of severity, by age group, by gender and by type of disability for each neighborhood. The population according to type and degree of disability corresponds to those officially recognized with the certificate of disability. This certificate is issued by the assessment and guidance teams at the care centers for people with disabilities of the Generalitat de Catalunya. The recognition is regulated by law [27] according to assessment tables that use the WHO classification. The datasets containing services have fields that allow for geo-tracking the corresponding facility.

Information about AIS and PADES resources (such as number of people assisted, areas of responsibility, or total population covered) has been found inside official reports from Observatori del Sistema de Salut de Catalunya in the Sociosanitari section [28].

After having organized and carried out a data exploration, we have developed our study using the following variables for each neighborhood: total population, population disabled, range age, type of disability, degree of disability, the average Family Income Available index (RFD acronym for its description in Catalan), the number of primary care centers, day care hospitalized centers, social centers, protected houses, elder hospitalized homes, elderly day care hospitalized centers and variable “All services”, which accounts for all the services in a neighborhood, i.e, the sum of primary care centers, day care hospitalized centers, social centers, protected houses, elder hospitalized homes, and elderly day care hospitalized centers.

### 2.2. Analysis Techniques

To reveal patterns and structure hidden in the data, we have applied the following techniques, which have been extensively applied to health care, as in [29,30,31,32]

#### 2.2.1. Cluster Analysis (ClA)

This is an unsupervised learning technique used to classify observations into a priori unknown groups called clusters [33]. The observations are thereby organized into an efficient representation that characterizes the data being sampled. Clustering methods are divided into two categories, hierarchical and non-hierarchical clustering. Hierarchical clustering groups the observations into groups, where the number of groupings that will appear is unknown. In non-hierarchical clustering, the number of groups is predetermined.

ClA is a natural technique to reveal discrete structures and come up with a profiling of Barcelona’s neighborhoods based on their disabled population, where homogeneous neighborhoods are placed in the same cluster trying to maximize the shared characteristics intra-group. In first place, we use a hierarchical clustering with the Ward Linkage method with squared Euclidean. To evaluate the robustness of the segmentation obtained, we have compared the results with two other two segmentation approaches. In particular, we have used another hierarchical clustering method, but now, with the ward linkage method, and the Euclidean distance; and a non-hierarchical clustering method where the number of clusters is predetermined and has been set to three.

#### 2.2.2. Multidimensional Scaling (MDS)

This technique provides a visual representation of distances or dissimilarities between sets of observations [34]. Observations that are more similar are closer together on the graph than objects that are less similar. From a slightly more technical point of view, MDS finds a set of vectors in p-dimensional space such that the matrix of euclidean distances among them corresponds as closely as possible to some function of the input matrix according to a criterion function called stress.

Normally, this technique is used to provide a visual representation of a complex set of relationships that can be scanned at a glance. For this reason, we have applied it in order to provide a 2D scatterplot of Barcelona’s neighborhoods based on the characteristics of the studied population.

#### 2.2.3. Correspondence Analysis (CA)

This is a technique for graphically displaying a two-way table by calculating coordinates representing its rows and columns. These coordinates are analogous to factors in a Factor Analysis [35], except that they partition the chi-square value used in testing independence instead of the total variance. This way, it takes a large table, and turns it into a easy-to-read visualization [36].

In this work, we use this method to determine how the placement of neighborhoods is affected by each categorical variable.

#### 2.2.4. Principal Component Analysis (PCA)

This is a technique for reducing the dimensionality of the variable space by representing it with a few orthogonal (uncorrelated) variables that capture most of its variability [37]. Thus, it transforms a number of (possibly) correlated variables into a (smaller) number of uncorrelated variables called principal components.

In the present study, we have applied PCA to visualize the positioning of the main medical areas of Barcelona in two dimensions, based on some of their features.

#### 2.2.5. Multiple Regression (MR)

This technique is an extension of simple linear regression. It uses several explanatory variables to predict the outcome of a response variable. Its goal is to model the linear relationship between the explanatory (independent) variables and response (dependent) variable [38]. When using MR, part of the process involves checking that the data to analyse can actually be analysed using MR.

We have modeled the relationships between population and health care services at the aggregated level of interest. To this end, we have tried various MR using different explanatory variables to choose the best subsets of independent variables maximizing the R-squared. We have analyzed the relationship between the population assisted by the PADES and different variable combinations among the type and degree of disability, the available services, or the economic factor.

## 3. Results

In this section, we present the assessment results for the elderly and disabled health care demand, the available health care services, and the relationship demand service for the city of Barcelona.

### 3.1. Elderly and Disabled Health Care Demand

As mentioned before, the demand assessment aims to profile Barcelona’s neighborhoods through their elder and disabled population. Barcelona has a total population of about 1,600,000 (see Appendix A for population distribution by neighborhood). Around 136,000 persons have a legally recognized disability, which represents 8.5% of the entire population.

**Figure 1:**
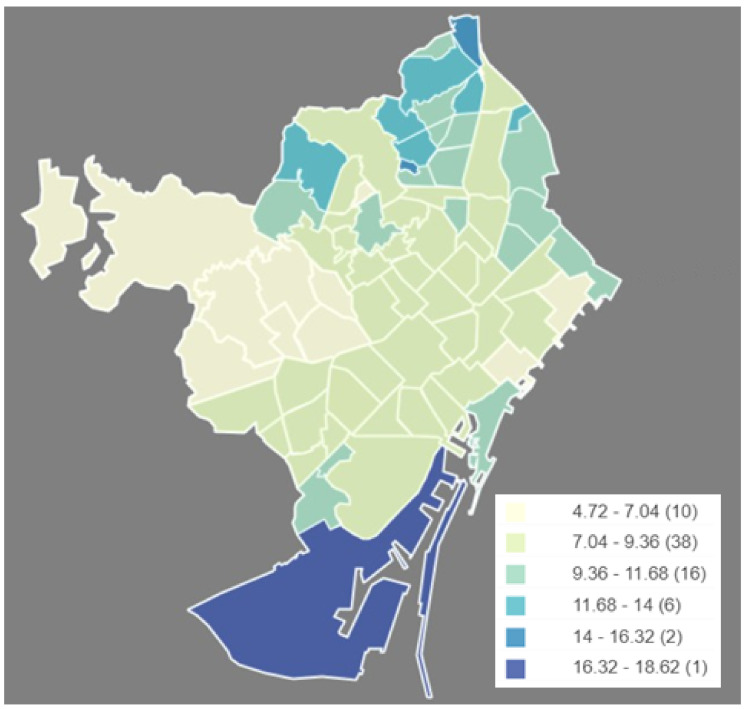
Neighborhood distribution for disabled people. (Own elaboration with Instamaps: www.instamaps.cat).

The Municipal Data Office [26] offers a data set of the disabled population showing type of disability, age range, sex, and degree of severity of disability for each neighborhood. Figure 1 shows the percentage of disabled population over the neighborhood total population. In general, we can see that the neighborhoods with higher relative percentage of disabled people are located in the north of the city. Exceptionally, there is the neighborhood la Marina del Prat Vermell (Ve) in the south, which has the highest percentage since it is the second least populated neighborhood, with 1150 inhabitants.

**Figure 2:**
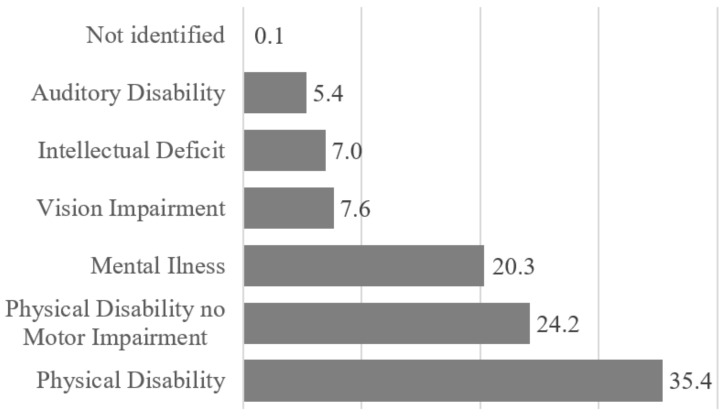
Types of disability distribution.

A preliminary analysis of the data set shows that the disability types are distributed with similar relative percentages across the neighborhoods, Physical disability being the most represented, and Auditory Disability the least represented (besides Not Identified). Figure 2 shows the type of disability distribution.

**Figure 3:**
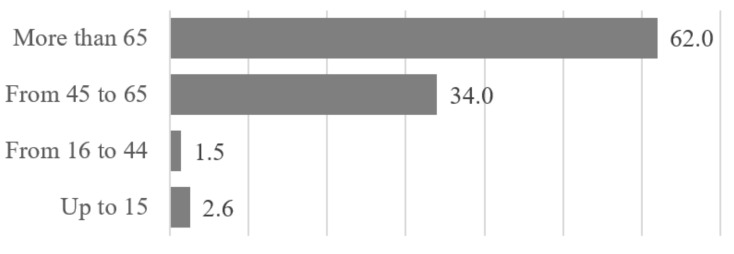
Disabled population age distribution.

The age of the population is also important when dealing with health care. The population of Barcelona mostly belongs to the 16 to 44 age range. However, in recent years, there has been a decline in youth population in favor of the elderly population. The ageing population is related to a health situation where chronic and complex pathology’s prevail and generate disability and dependence. Figure 3 illustrates how disabled population is distributed according the age range. As expected, most of disabled people are in advanced age, e.g., 62% of people are more than 64 years old.

**Figure 4:**
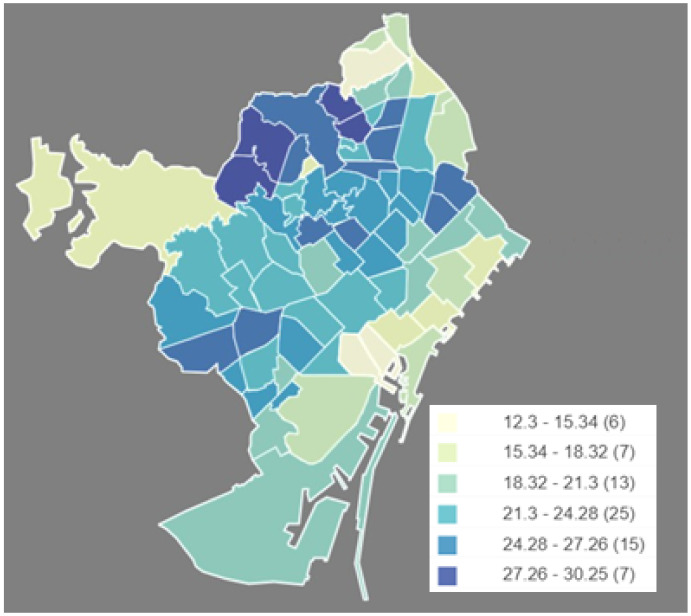
Neighborhood distribution for population older than 64 years old. (Own elaboration with Instamaps: www.instamaps.cat).

Given the relationship between the elderly and disabled population, Figure 4 shows the percentage of people older than 64 years old over the total population in each neighborhood. We can observe that, in general, neighborhoods with high values for people with disabilities tend to be among those with more elderly people. Thus, again, we can see high percentages of elderly population living on the north neighborhoods.

#### 3.1.1. Mapping the Neighborhoods

The impact of each categorical variable and a 2D mapping of the neighborhoods based on those variables can be captured by applying the MDS and CA techniques.

The CA based on all the categorical variables explains 66.8% of the variance suggesting that there may be other factors determining the neighborhoods’ placement. The most significant categories for the analysis are related to the age range. There is a positive correlation between vision impairment and auditory disability, but they are not significantly affected.

**Figure 5:**
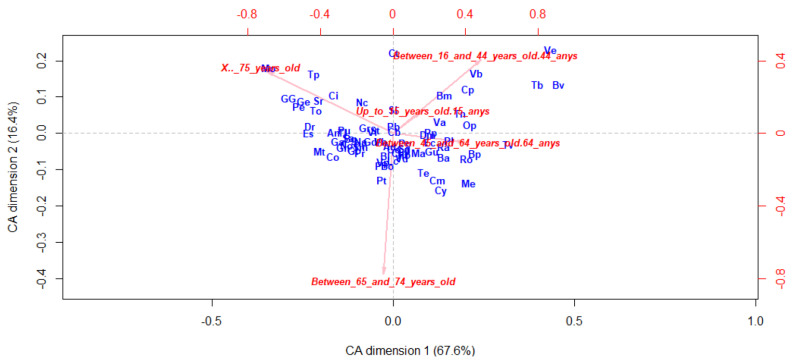
Correspondence Analysis by neighborhood based on age range.

**Figure 6:**
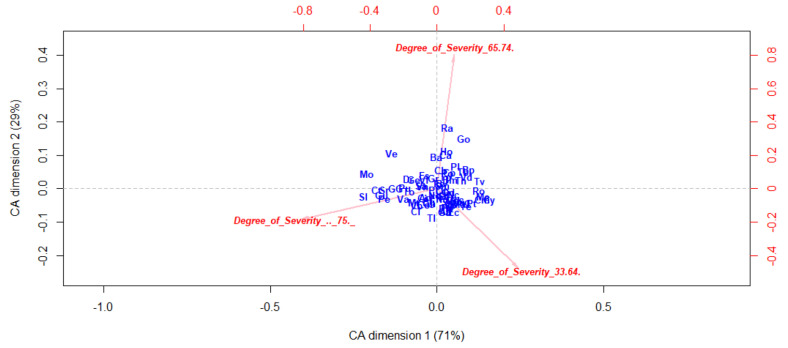
Correspondence Analysis by neighborhood based on degree of severity.

**Figure 7:**
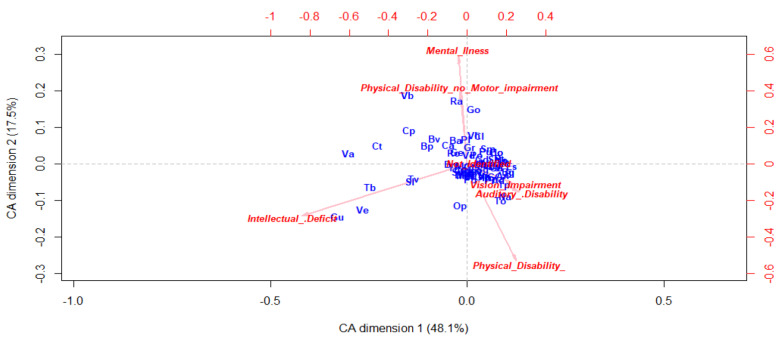
Correspondence Analysis by neighborhood based on disability type.

In order to represent a more insightful way to inspect the needs of each neighborhood and detect which characteristics are specific to each population, separate CA analysis have been carried out. Figure 5, Figure 6 and Figure 7 show the byplot for the CA based on age range, degree of severity and disability type, respectively. The neighborhoods distribution on the plots gives us an idea of how similar or distinct they are, as well as the significance of the characteristics. Thus, the scattered and low density of the neighborhoods points on Figure 5 brings up that the population’s age distribution changes significantly across neighborhoods.

Figure 6 presents a higher density of neighborhoods points, suggesting an homogeneous distribution regarding the degree of severity among the different neighborhoods. Note that the neighborhood of El Raval (Ra) is slightly separated, indicating a significant percentage of people with a medium level of disability.

Regarding the disability type, in Figure 7 we can see a dense zone of points that reveals that there are a significant number of neighborhoods with similar conditions. However, we can observe that there are a few number of neighborhoods with significance of intellectual deficiency (Gu, Ve, Tb, Sv), mental illness, or physical disability without motor impairment (Ra, Go, Vb, cp, Bv, Bp).

#### 3.1.2. Segmentation of the Neighborhoods through Disabled Demographics

In order to obtain a segmentation of the neighborhood based on their insights, we have applied CLA. We have implemented a row-profiling by percentage approach in which neighborhood counts for each variable are divided by the total disabled population in that neighborhood. This allows us to ignore the scale effect, prioritizing instead the structural composition. Such clustering allows neighborhoods of different sizes to be placed in the same group if their populations have similar marginal distributions.

#### 3.1.3. Hierarchical Clustering

We have applied the clustering technique taking into account the marginal distributions in percentage across every neighborhood for the following categorical variables: type of disability, age range, severity grade of disability and sex. The values for every variable were standardized by using the chi-square standardization, which is particularly effective to handle data that are percentages or proportions.

**Figure 8:**
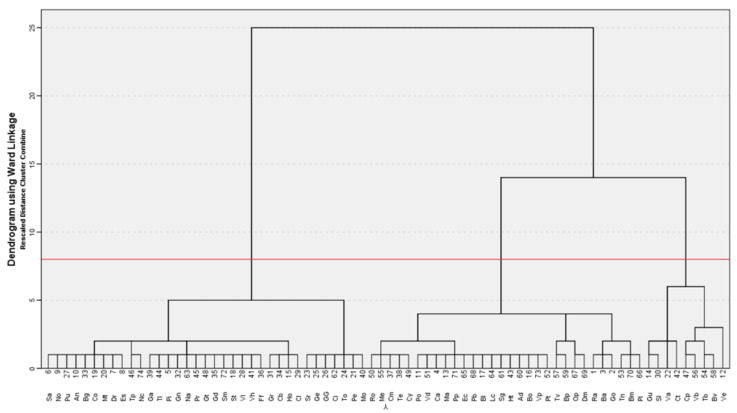
Neighborhoods dendrogram based on their disabled population.

As mentioned before, in order to decide which neighborhoods should be combined, we have applied the ward linkage method with squared Euclidean distance to prioritize the minimization of the total within cluster variance. This combination of linkage criterion and metric is very popular and appreciated for its robustness. The sequence of merging clusters can be seen in the dendrogram of Figure 8.

For deciding the number of clusters to use in the neighborhoods’ segmentation, the objective is to maximize homogeneity intra cluster and heterogeneity inter clusters. The distance provided on the vertical axis of the dendrogram displayed above indicates the resemblance between neighborhoods. Thus, the number of clusters can be determined by the number of vertical lines which are intersected by the horizontal line that cuts the longest vertical line. Since longer vertical lines in the dendrogram imply more distance (less resemblance) between those clusters, the longest vertical line identifies less similar neighborhoods. In the case of our interest, three seems to be the optimal choice. This result is consistent with other methods for determining the optimal number of clusters. In that sense, studying the distance increase inter cluster by each additional cluster through a Scree Plot, it can be seen, also, that the last number of cluster that implies a significant marginal decrease in distance corresponds to three clusters. Adding an extra one would mean having two clusters whose relative distance would not be significant implying higher risks of misclassification. So, there is not a justification for having a fourth cluster or more.

The details of the clusters can be extracted from Figure 8. Starting from the left, the first cluster is the biggest one with 35 neighborhoods, the second cluster has 30 neighborhoods, and the third cluster is the smallest with nine neighborhoods. Heterogeneity inter clusters is maximum between the first one and the other two, and minimum between the second and the third clusters. At the intra cluster level, the homogeneity is highest in the second group and lowest in the third cluster.

**Figure 9:**
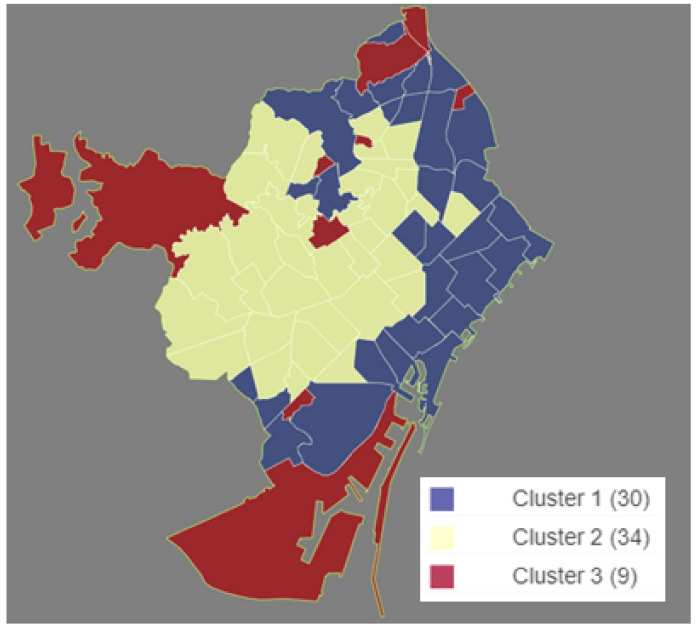
Neighborhoods’ segmentation by clusters. (Own elaboration with Instamaps: www.instamaps.cat).

The geographic distribution of the neighborhood’s segmentation can be seen in the map of Figure 9. Note that, in general, neighborhoods belonging to the same cluster are adjacent, except for those belonging to Cluster 3, even though the geographical proximity was not taken into account during the segmentation process.

#### 3.1.4. Robustness Validation

Table 1 shows and compares in a cross-tabulation the segmentation results obtained previously with those computed to evaluate the robutness. In general, the clusters hold with the new segmentation results, even though there are some misclassifications. Using the ward linkage method with Euclidean distance, only two neighborhoods are misclassified, shifting from Cluster 3 to Cluster 2 which are the most similar pairwise. When applying the K-means algorithm, the segmentation obtained is slightly different, with four more misclassifications. In addition to the two neighborhoods shifting from Cluster 3 to 2, there are two neighborhoods shifting from Cluster 3 to 1 and two neighborhoods shifting from Cluster 1 to 2.

Having consistency among the three segmentations means that the segmentation is independent from the specific implementation and, therefore, robust. The previous analysis results show the “acceptable” behaviour of the implementation, where the clusters mostly remain consistent. A more in-depth study, which includes new features to help characterize neighborhoods, would be useful to improve the neighborhood classification model, thus obtaining a more reliable segmentation, especially of the most heterogeneous group, Cluster 3.

### 3.2. Health Care Service

After analyzing the demand, this section covers the offer of health care services available in the city of Barcelona.

#### 3.2.1. AIS—Integral Health Areas

The AIS were defined considering geographical elements and patients’ behavioral patterns, in order to improve coordination among sanitary services and to facilitate patients’ follow-ups through a comprehensive health care framework. In that sense, four areas cover the city: AIS Nord, AIS Esquerra, AIS Dreta and AIS Litoral (see Appendix B).

**Figure 10:**
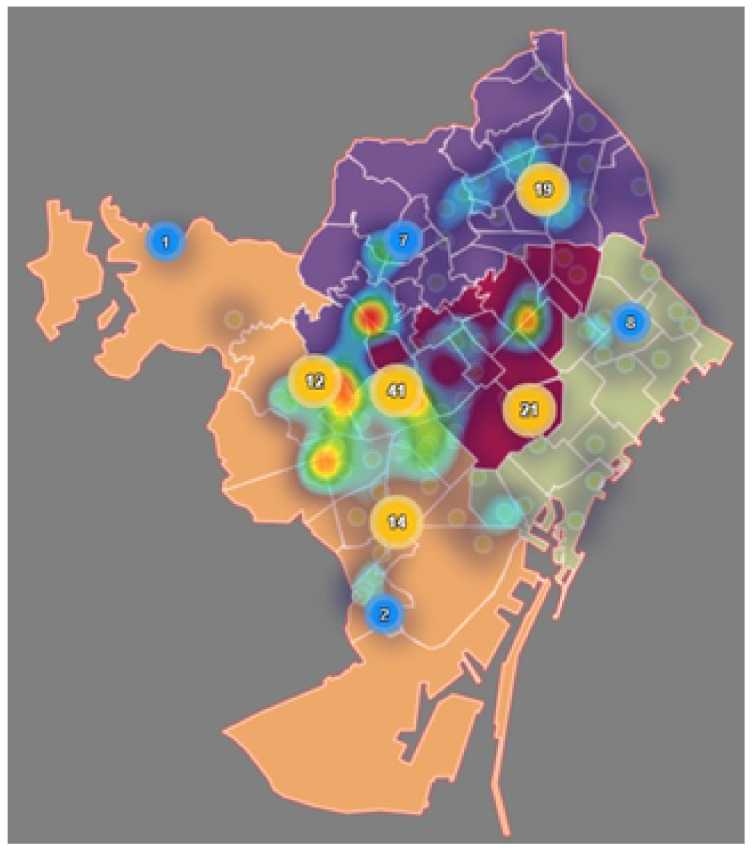
Distribution and density of primary care centers by AIS. (Own elaboration with Instamaps: www.instamaps.cat).

Figure 10 reports the distribution of primary care access points (mainly CAPs and hospitals) across AIS, highlighting the areas with a higher density of clinics, which correspond to the center of the city (AIS Esquerra and AIS Dreta).

#### 3.2.2. PADES—Home Care Program

PADES is a home health care and personal assistance program considered as a reference model in the home health care domain [39]. PADES are allocated to cover the population of several ABS (see Appendix B).

**Figure 11:**
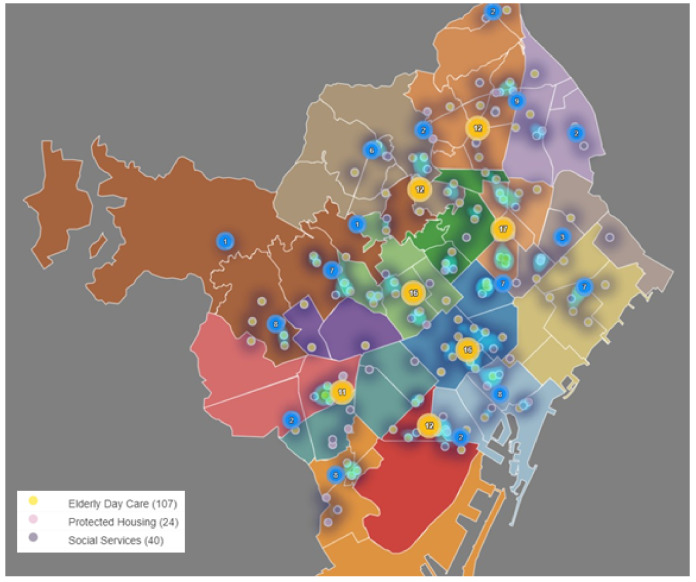
Distribution of elderly day care, protected housing and social services by PADES area. (Own elaboration with Instamaps: www.instamaps.cat).

Figure 11 shows how social services, elderly day care hospitalized centers and protected housing centers are distributed in the PADES areas. It can be seen that most of the services are located in the North-East of the city, corresponding with the AIS Nord and AIS Dreta.

### 3.3. Relationship between Demand and Service

In this section, we try to point out meaningful links between the city’s elderly and disabled population heath care demand and the available health care services by analyzing what drives coverage across the different territorial areas. The analysis is performed from the lowest to the highest management unit: neighborhoods, PADES areas and AIS areas.

#### 3.3.1. Assessment by Neighborhoods

In order to look for quantitative relationships between services and demand population features, it is suitable to build scatter plots of the different variables involved.

**Figure 12:**
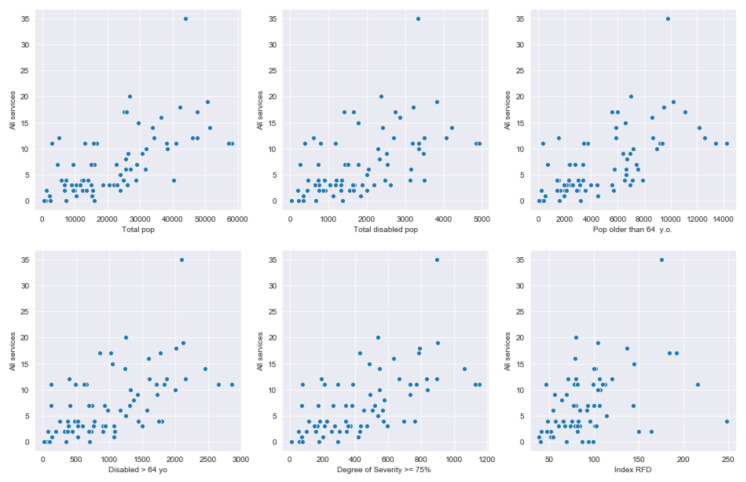
Scatter plots of total number of services and relevant explanatory variables.

Scatter plots of Figure 12 suggest that values for “All services” are quite scattered across the plane, there is a certain amount of dispersion and no very precise linear relationships can be detected. Nevertheless, the increase in people, in general, is accompanied by an increase in the number of services. The Family Income Available index (RFD acronym for its description in Catalan) seems to have limited influence on the number of services. Overall, the graphs indicate that other factors are at play in determining the number of services of a neighborhood. For instance, the geographical complexity of a city was not took into account, but most likely it has a very significant effect in the location of the services.

A deep analysis in which several features combinations were tried and checked for multicollinearity brings out that the variables on hand do not fully capture the relationship with the number of services in a neighborhood. In that sense, the model with the highest R-squared was obtained by using the RFD index and the disabled population with a degree of severity higher than 75% as predictors. It has a goodness of fit about 50%.

**Figure 13:**
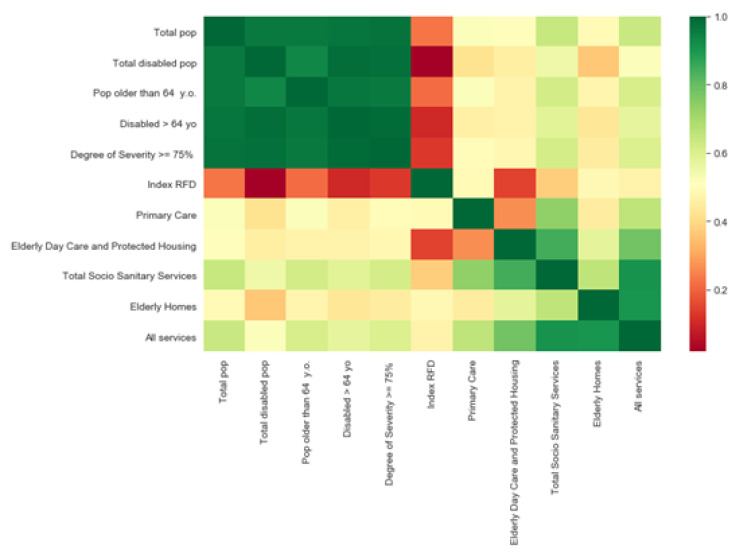
Correlation matrix between variables at a neighborhood level.

In order to have a better understanding of the insight in the neighborhoods, it is worthwhile to visualize the relationships between each independent variable, looking at the correlation matrix in Figure 13. Note that the RFD index is negatively correlated with disabled demographics, especially with the total disabled population. This indicates that richer neighborhoods tend to have a smaller disabled population. This leads to the premise that socioeconomic inequalities may affect population’s health. In that sense, it can be seen that the total number of services is slightly negatively correlated with the RFD index. Thus, a greater number of the available resources are located in the poorest areas, which are the areas that need the most support.

#### 3.3.2. Assessment by PADES Area

This section studies the causal relationships able to explain the number of people assisted by a certain PADES area in relation to its demographics and territorial characteristics.

The PADES areas are different in terms of covered population size and distribution. In order to study the number of persons assisted by each PADES area (called “Pades assisted” variable), the neighborhood population features are grouped according to PADES areas. Thus, for instance, the average RFD index of a PADES area is obtained by computing the average between the RFD index of those neighborhoods that belong to that PADES area.

**Figure 14:**
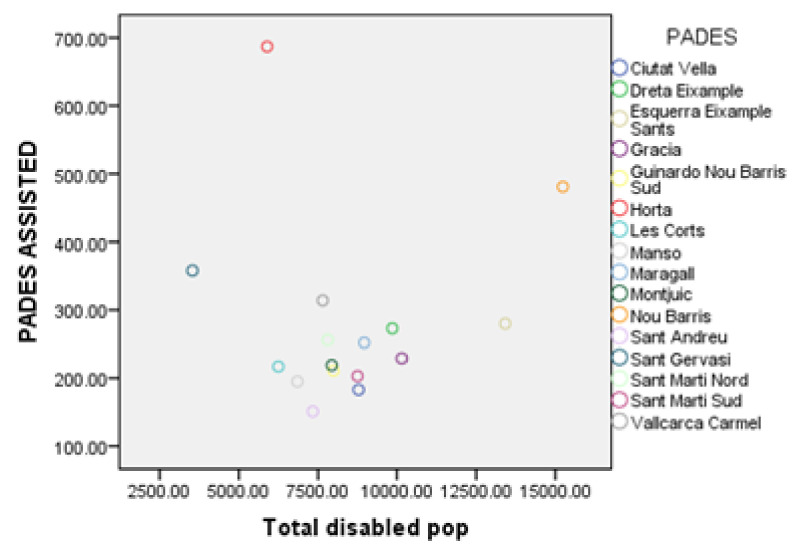
Number of people assisted by PADES area respect total disabled population.

**Figure 15:**
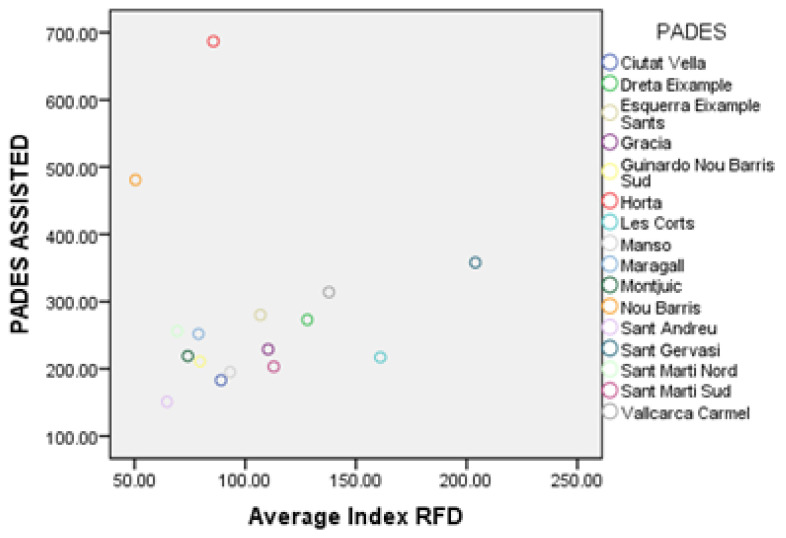
Number of people assisted by PADES area respect the average RFD index.

The application of the multiple regression technique (see Appendix C) shows that both the total disabled population and the RFD index are main predictors for the number of persons assisted (“Pades assisted” variable). The scatterplots of these relationships (see Figure 14 and Figure 15) reveal that the number of people assisted has a positive correlation with the total disabled population, as well as with the average RFD index. However, in both features, there seem to be some anomalous data points. In particular, for the total disabled population, the PADES of Sant Gervasi and Horta lie far from the trend line. In the meantime, for the RFD index, the PADES of Nou Barris and Horta is the one placed outside the trend line.

The regression’s results also provide further insights on the PADES allocation, bringing out that the number of persons with physical disability is a meaningful variable for the number of assisted persons. Considering that physical disability is often associated with motor impairment, it makes sense that this type of disability drives the PADES coverage, since people affected are most likely to need support to carry out their daily routines. As expected, another predictor is the number of social and palliatives services in the area. Having a higher number of services available in the area affects positively the number of assisted people, since the PADES teams of the area have at hand more resources to exploit.

#### 3.3.3. Assessment by AIS

This section studies the relationship between the service available in the AIS areas and the population in those areas. In order to have properly balanced AIS areas, the population characteristics and territorial features must be as homogeneous as possible among the different areas. The same variables so far employed were also used, where the totals for each AIS area were computed by summing the observations of each PADES area allocated to the given AIS area. From the PCA carried out on the standardized dataset, we can extract that AIS Dreta and AIS Esquerra display similar behaviours, while AIS Litoral Mar and Nord are clearly differentiated.

**Figure 16:**
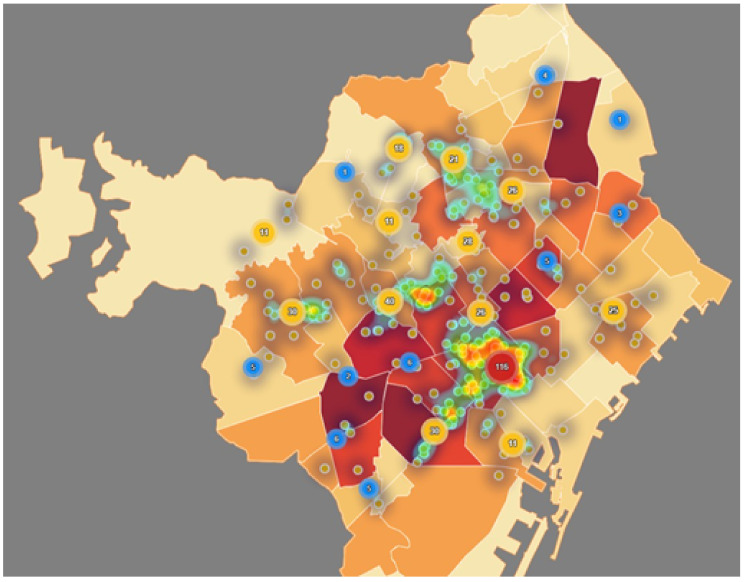
Elderly care homes vs. elderly distribution. (Own elaboration with Instamaps: www.instamaps.cat).

**Figure 17:**
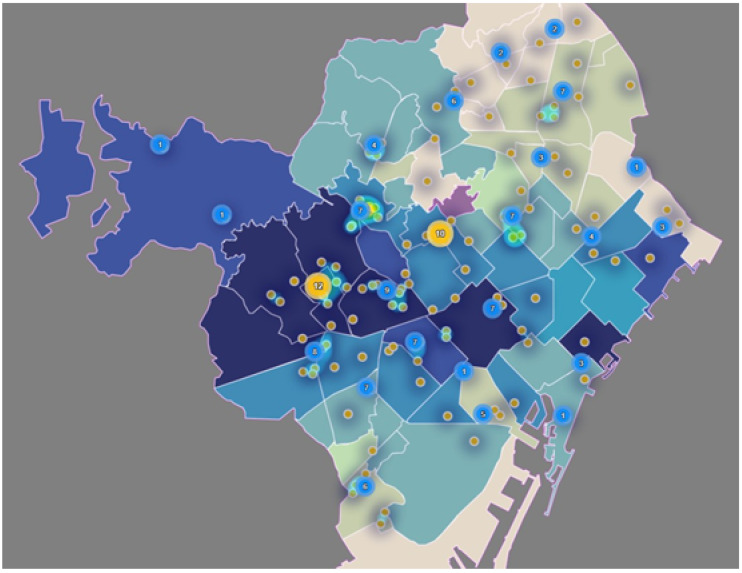
Primary care centers distribution vs. RFD index. (Own elaboration with Instamaps: www.instamaps.cat).

Figure 16 and Figure 17 conclude this section, providing two maps that relate demand and service offer. On one hand, Figure 16 shows that elderly care hospitalized homes are distributed accordingly to elderly population distribution (in absolute value), which is highest in Dreta and Esquerra AISs. Note that a very dense cluster is located in a strategically central position in the AIS Dreta. On the other hand, Figure 17 focuses on the distribution of primary care services in relation to the RFD index. As already pointed out, Dreta and Esquerra AISs are the wealthiest. A higher density of primary care services can be observed in these areas. Although, in the AIS Nord there is a significant population (both disabled and not), the services density is clearly less.

## 4. Discussion

The city’s health care system involves several lines of action: primary care, emergency care, hospital care, walk-in care, intermediate care, social healthcare and mental healthcare. Intermediate care is defined by the British Geriatrics Society [40] as “those services that do not require the resources of a general hospital, but are beyond the scope of the traditional primary care team”. According to [41], intermediate care can include substitutional care and care for people with complex needs. Thus, social healthcare and intermediate care will be specially relevant for this study.

This study presented an assessment of the links between disabled population and health care services in Barcelona and provided evidence that demand’s coverage takes into account territorial and demographics needs. The analysis on elder and disabled population across neighborhoods evidences a regularity in its distribution. In fact, relative composition varies on a limited range, indicating that features taken into account are likely following a normal distribution. Still, there are meaningful differences among neighborhoods to apply a classification model. Indeed, the clustering model revealed that the differences in composition are relevant and useful for assessing the needs of the neighborhoods. In particular, the segmentation led to the definition of three neighborhood classes: the first one is characterized by a population with a lower severity degree of disability, in the second class, the percentages of older people and cases of motor disability are higher, and the third is differentiated by a younger demographic group and more widespread mental illnesses. The robustness validation indicated that elements from the last class are the most difficult to classify.

Barcelona’s health care system has multiple organizational layers with different competences and scopes; AIS and PADES are the most important ones. The AIS aggregate different healthcare providers and assure coordination among them by defining guidelines and targets for the services in the territory. Primary health care is the first and major face-to-face access point to healthcare for the citizens. In fact, the rest of the services, besides emergency care, are accessed through the Primary Health Care Center (CAP), which then re-directs the patient to a suitable healthcare service. CAPs are spread over Barcelona and oversee one or more “Àrees Bàsiques de Salut” (ABS, Basic Health Areas), which are the elementary territorial unit of medical competence (see Appendix B). Each ABS has, in turn, a primary care team formed by general practitioners, pediatricians, dentists, nurses, nursing assistants, social workers and non-health personnel, all in charge of providing health care services to the population of a given ABS. PADES teams are formed by experts in palliative care and in the care of people with advanced chronic disease, professionals in the fields of medicine, nursing, social services and psychologists. The program’s objective is to deliver straight to the patients’ homes the care services they need, favoring the maximum conditions of autonomy and comfort.

From the decision making point of view, proper resource allocation is quite relevant, since it can reduce medical expenses and also enhance patient satisfaction. Uncertainty and dynamism are two additional issues that clearly hinder the scheduling and management of the home health cares compared with the traditional hospitalization. Thus, Ref [42] argues that home health care has unique features and highlight the fact that its services are affected by dynamic environments and high randomness created by uncertain factors, such as variable demand, stochastic traveling time of the workforce and variable service time. Last minute changes occur and staff allocation has to quickly adapt, especially for time critical care services.

In order to consider organizational improvements, we have compared the neighborhood’s segmentation with the PADES ans AIS areas. The fact that, in general, neighborhoods belonging to the same cluster are adjacent, even though the geographical proximity was not taken into account, simplifies the implementation of those upgrades. Furthermore, the segmentation could be used to assess the neighborhood requirements and be used by the Sanitary Consortium of Barcelona to develop specific guidelines for operations adapted to each cluster. For instance, the PADES teams in each cluster area may be made up of professionals who address the real needs of the population in the area. Thus, the second cluster would have a bigger workforce and would be more used at working with older demographics, while teams of the third cluster would have specific expertise in mental illnesses.

The Sanitary Consortium of Barcelona is intensifying its commitment to home health care as highlighted in its Health Plan, which mainly affects the PADES program. The study shows that, on average, about 8% of each PADES area population is disabled, with peaks of 10% in Montjuic and Nour Barris, and the lowest value in Sant Gervasi with a 5%. The PADES program provides significant support to the disabled population; about 4% of the disabled population receives assistance from a PADES team. However, the coverage is not completely homogeneous, Horta and Sant Gervasi offer a higher coverage of 11% and 10%, respectively; while the PADES located in Dreta and Esquerra AIS areas have a coverage of 3%.

AIS Dreta and AIS Esquerra display similar behaviors. These areas correspond with the wealthiest and most populated AISs. Their average RFD index are similar, with Esquerra leading by about 10 points. Their older segments are also similar, both around 109,000 persons. In terms of disabled population, AIS Esquerra has a slightly higher number, around 38,000 people compared to 37,000 in AIS Dreta. However, AIS Dreta leads the total health care services, maybe due to its more central position in the urban landscape. However, even so, both areas have about the same people assisted by their PADES team, nearly 1000 persons.

AIS Litoral Mar has the lowest values of disabled population, of older than 64 years old population, and, in general, of the total population. Accurately, the number of persons receiving assistance from PADES is also the lowest one. Moreover, it has less than half the number of Dreta’s health care services.

AIS Nord is the area with the lowest RFD index and the greatest percentage of elderly population. Although the number of population older than 64 years old is lower than in Dreta and Esquerra AISs, the ratio between the old population and the total population is the highest. Analogous, in terms of disabled population, the numbers are comparable with those from Dreta and Esquerra AISs. However, the difference in the total population lets in a significantly higher percentage in disabled population. This can be explained by the elderly population proportion. It is very likely that this distribution explains the fact that AIS Nord has the highest value for assisted people (around 1600). In line with this, there is a significant number of health care services in the area, showing that resources were allocated to deal with the situation.

The coverage rate can be explained through the area’s demographics. In particular, coverage is driven by a mixture of factors: motor impairment, severity of disability, number of already existing services in the area and RFD index. The study states that the RFD index of an area is negatively correlated with its total number of disabled people, suggesting that socioeconomic inequalities have consequences on a person’s health. Furthermore, the RFD index of an area has a controversial effect on the area’s health care services: on average a higher RFD do not imply a higher number of health care services, but both the total number of people assisted by PADES and the number of primary care centers seems to be positively affected by an increase in the RFD.

Despite the positive correlation between the number of people assisted and the total disabled population or the average RFD index, the Horta area, without a significant number of disabled population nor a high RFD index, has the highest number of assisted population in the city. A possible explanation can be the fragile demograpics of the area with high percentages of elderly people and people with a high degree of severity, which are most likely to need assistance. Furthermore, geographically this area of Barcelona is not very accessible, and as a consequence, people with motor impairment can find it hard to move around, needing more assistance.

Sant Gervasi’s is placed outside on the relationship trend with the total disable population. This can be explained by the fact that corresponds to the wealthiest PADES area in Barcelona, and therefore, it is more likely to have access to a better home health care service even though it is the least populated PADES in the city. On the other hand, there is the Nou Barris PADES, with the lowest average RFD index, meaning that its population is the most indigent in Barcelona. Nonetheless, the area is still covered from the PADES program, due to its high number of disabled population.

The PADES program provides significant support to the disabled population and is an important asset of the Barcelona health care system. This is in line with the strategy outlined in the Barcelona Health Plan which establishes that efforts be made to reduce health inequalities. In that sense, lately the Sanitary Consortium of Barcelona is trying to reduce inequalities in health by placing care services in poor areas.

## 5. Conclusions

In this paper, we present a descriptive-analytics-based methodology to provide important insights on demographic and territorial aspects needed for future planning. We have conducted a study to assess the health care demand of elder and disabled population and the available health care services in Barcelona. We provide an analysis of the relationship between the health care services for the mentioned population and the current offer of services. The study provided evidence that demand’s coverage takes into account territorial and demographics needs and that the health care resources are placed in accord with the elderly and disabled populations distributions. Furthermore, the study pointed out that the different AIS in the city have distinguishable demographics characteristics. The results obtained from the study are greatly useful for the Barcelona Sanitary Consortium, since it allows them to tailor health care services and resources to meet the particular needs of each area. Future research can explore specific recommendations of location and reallocation of services in order to adapt the health care infrastructure to meet the needs of the population.

## Figures and Tables

**Table 1:** Cross-tabulation showing cluster placement.

		Ward Method with Euclidean Distance			K-Means		
		Cluster 1	Cluster 2	Cluster 3	Cluster 1	Cluster 2	Cluster 3
Ward method	Cluster 1	35	0	0	33	2	0
with Square	Cluster 2	0	30	0	0	30	0
Euclidean distance	Cluster 3	0	2	7	2	2	5
Total		35	32	7	35	34	5

## Abbreviations

The following abbreviations are used in this manuscript:

ABS	Basic Health Areas
AIS	Integral Health Areas
CA	Correspondence Analysis
CAP	Primary Health Care Center
CLA	Cluster Analysis
MDS	Multidimensional Scaling
MR	Multiple Regression
PADES	Home Care Program
PADRIS	Public Data Analysis for Health Research and Innovation Program
PCA	Principal Component Analysis
RFD	Family Income Available
